# Unprecedented Therapeutic Potential with a Combination of A_2A_/NR2B Receptor Antagonists as Observed in the 6-OHDA Lesioned Rat Model of Parkinson's Disease

**DOI:** 10.1371/journal.pone.0114086

**Published:** 2014-12-16

**Authors:** Anne Michel, Patrick Downey, Jean-Marie Nicolas, Dieter Scheller

**Affiliations:** 1 Neurosciences TA Biology, UCB BioPharma SPRL, Braine l'Alleud, Belgium; 2 Non-Clinical Development, UCB BioPharma SPRL, Braine l'Alleud, Belgium; Prince Henry's Institute, Australia

## Abstract

In Parkinson's disease, the long-term use of dopamine replacing agents is associated with the development of motor complications; therefore, there is a need for non-dopaminergic drugs. This study evaluated the potential therapeutic impact of six different NR2B and A_2A_ receptor antagonists given either alone or in combination in unilateral 6-OHDA-lesioned rats without (monotherapy) or with (add-on therapy) the co-administration of L-Dopa: Sch-58261+ Merck 22; Sch-58261+Co-101244; Preladenant + Merck 22; Preladenant + Radiprodil; Tozadenant + Radiprodil; Istradefylline + Co-101244. Animals given monotherapy were assessed on distance traveled and rearing, whereas those given add-on therapy were assessed on contralateral rotations. Three-way mixed ANOVA were conducted to assess the main effect of each drug separately and to determine whether any interaction between two drugs was additive or synergistic. Additional post hoc analyses were conducted to compare the effect of the combination with the effect of the drugs alone. Motor activity improved significantly and was sustained for longer when the drugs were given in combination than when administered separately at the same dose. Similarly, when tested as add-on treatment to L-Dopa, the combinations resulted in higher levels of contralateral rotation in comparison to the single drugs. Of special interest, the activity observed with some combinations could not be described by a simplistic additive effect and involved more subtle synergistic pharmacological interactions. The combined administration of A_2A_/NR2B-receptor antagonists improved motor behaviour in 6-OHDA rats. Given the proven translatability of this model such a combination may be expected to be effective in improving motor symptoms in patients.

## Introduction

The progressive loss of dopaminergic neurons from the substantia nigra pars compacta (SNc) leads to striatal dopamine (DA) deficiency and the emergence of the cardinal motor symptoms of Parkinson's disease (PD): bradykinesia, resting tremor, rigidity and postural instability [1]. While DA replacement therapy is the gold standard for treating patients with PD, the use of L-Dopa or DA agonists is associated with motor complications such as dyskinesia, dystonia, wearing-off and on/off phenomenon [2]–[4].

The emergence of significant motor complications associated with dopaminergic agents and the fact that such side-effects can become severely disabling highlights the need to develop innovative therapies able to circumvent the severe complications associated with deleterious neuro-adaptations resulting from dopaminergic neurodegeneration and pulsatile dopaminergic therapy [5], [6]. As direct modulation of the dopaminergic system eventually leads to serious side effects and, in the long term, becomes ineffective, significant effort has been invested to find non-dopaminergic targets. Two targets which have shown great promise in preclinical disease models are the adenosine A_2A_ receptor and the NR2B subunit of the NMDA receptor.

Adenosine 2A (A_2A_) receptors are abundant in the striatum, of both rodent and human brains [7] and are specifically expressed in GABAergic striatopallidal neurons (i.e. indirect output pathway) [8]. Within these neurons they co-localize with dopamine D2 receptors [9] and are able to form A_2A_-D_2_ heterodimeric complexes [10]. Mechanistically, activation of the G_S_ coupled A_2A_ receptors will antagonize signaling of the G_i_ coupled D2 receptor at the level of cAMP, while stimulation of the A_2A_ receptor reduces the ability of dopamine to bind to the D2 receptor by means of an intra-membrane A_2A_–D2 receptor interactions [11]. The observation that A_2A_ receptors functionally oppose the actions of D_2_ receptors on GABAergic striatopallidal neurons, led to the hypothesis that A_2A_ antagonists could enhance the activity of dopaminergic agents in alleviating parkinsonian motor symptoms [12] and also act by themselves to reduce the over-activity of the indirect pathway and the severe motor inhibition associated with it [13]. In rodent or primate models, when A_2A_ antagonists are given alone (i.e. as monotherapy) to severely DA-depleted animals they show only marginal activity [14]–[16], however, they are able to significantly potentiate dopaminergic treatment [17]–[21]. In the clinic, when the A_2A_ antagonist Istradefylline was given as monotherapy (i.e. without L-Dopa) to *de- novo* PD patients, it did not produce statistically significant benefits [22]. However, when combined with L-Dopa, Istradefylline, and other A_2A_ antagonists, demonstrated significant efficacy [23]–[25]. In fact, Istradefylline is now approved in Japan as add-on treatment to L-Dopa because of its ability to counteract wearing-off phenomena in fluctuating PD patients [26].

Striatal dopamine depletion is also associated with over activation of the glutamatergic NMDA receptors [27]. A number of studies have examined the efficacy of NMDA antagonists in animal models of PD. These studies showed that NMDA receptor blockade alleviates the parkinsonian motor symptoms, augments the effectiveness of dopaminergic therapy and can even prevent or reverse the induction of involuntary movements induced by L-Dopa [28], [29]. However, non-selective NMDA receptor antagonists have limited therapeutic value due to mechanism based side-effects. Accordingly, the modulation of specific receptor subtypes might provide a better alternative to modulate glutamatergic input to the basal ganglia [28]. In particular, NR2B receptor antagonists have been proposed as promising alternatives for the treatment of the motor symptoms of PD [30]–[32] and have been shown to be effective in alleviating experimental parkinsonism in both rodent and non-human primate models of PD [33]–[36]. NR2B antagonists have been shown to potentiate the therapeutic effect of L-Dopa [34], [37], [38] and to be effective in reducing L-Dopa-induced dyskinesia [33], [39]. In both rat and human brains, NR2B receptors are expressed throughout the brain, with high expression in the cortex, hippocampus, striatum, thalamus and olfactory bulb [31]. In situ hybridization studies identified intense NR2B signals in all striatal neuronal populations in human brains [40]. While the selective NR2B antagonist, CP-101,606 demonstrated efficacy in counteracting L-Dopa-induced dyskinesia in a randomized double-blind placebo-controlled clinical trial [41], a single clinical study with the selective MK-0657 gave negative results when administered as monotherapy [42].

There is an increasing body of evidence which suggests that the NMDA and A_2A_ receptors interact, at least, within the striatum. Indeed, it has been suggested that these two receptors share a common intracellular signaling pathway, whereby NMDA receptor signaling increases the activity of the A_2A_ receptors [43]. Therefore the efficacy of an A_2A_ antagonist should be enhanced by the co-inhibition of the NMDA receptor. Interestingly, activation of the G_s_ coupled A_2A_ receptor will increase protein kinase A (PKA), and PKA is known to increase the functionality of the NMDA receptor [44]–[46]. Therefore, within the striatum, the A_2A_ and NMDA receptors may act to mutually stimulate each other, suggesting that inhibiting both receptors could provide significant benefit.

The objective of this study was to explore the potential therapeutic benefit of combining A_2A_ and NR2B antagonists for the treatment of the motor symptoms of PD. The efficacy of six different combinations was assessed in a classic preclinical model of PD, i.e. the unilateral 6-OHDA-lesioned rat model [47], [48]. To explore the effect of this new potential treatment on motor symptoms, A_2A_/NR2B receptor antagonist combinations were tested without L-Dopa (*monotherapy*) and as adjunctive treatment with a low active dose of L-Dopa (*add-on therapy*). The A_2A_ and NR2B receptor antagonist drugs used were selected on the basis of their specific selectivity and affinity for the two respective receptors (see **Table 1** and **Table 2**). To explain superior level of motor activity, statistical analysis, three-way mixed ANOVA, first, aimed at investigating the overall effect of the A_2A_ and NR2B receptor antagonists and second, explored the effect of their interaction. The nature of the interaction, whether additive or synergistic was evaluated and any pharmacokinetic interaction using two combinations as examples were ruled out.

**Table 1:** Adenosine A_2A_ antagonists and their affinity for the receptor: in vitro binding.

A_2A_ antagonists	Pharmaceutical Company	*Ki* a A_2A_	*Ki* A_1_	*Ki* A_2B_	*Ki* A_3_	References
**Istradefylline**	Kyowa Hakko Kirin Co Ltd	12	841	>10,000	4,470	[71]
**Sch-58261**	Schering-Plough	0.6	287	5,011	>10,000	[71], [72]
	Merck & Co Inc	5	725	1110	1200	
**Preladenant**	Merck & Co Inc	1.1	>1000	>1,700	>1,000	[73] [71]
		0.9		>1000	>1000	
**Tozadenant**	Biotie Therapies	4.9	1,320	ND	ND	[71]

a Ki is expressed in nM (rat or human).

ND: no data available.

**Table 2:** NR2B antagonists and their affinity for the receptor: in vitro and functional binding.

NR2B antagonists	Pharmaceutical Company	NR2B	NR2A	NR2C	Assays	References
**Merck 22**	Merck & Co Inc	*Ki* = 0.88 nM	ND	ND	In vitro Binding	[49]
**Radiprodil**	Gedeon Richter	IC_50_ = 3–10 nM	IC_50_>10 µM	ND	In vitro Binding	[69]
	UCB					
**Co-101,244**	Parke-Davis	IC_50_ = 0.026 µM	IC_50_ = 230 µM	IC_50_ = 590 µM	TEVC recording	[33], [74]
	Pfizer	IC_50_ = 0.043 µM	IC_50_>100 µM	IC_50_>100 µM		
		IC_50_ = 4 nM			In vitro binding	[75]

ND: no data available.

## Materials and Methods

### Animals and ethic statement

All animal experiments were performed according to the Helsinki declaration and conducted in accordance with the guidelines of the European Community Council directive 86/609/EEC. The ethical committee from UCB BioPharma SPRL (LA1220040 and LA2220363) approved the experimental protocols. Male Sprague-Dawley rats (Janvier, France) were housed in cages (4 rats per cage) for one week before experimentation. They were kept on a 12∶12 light/dark cycle with light on at 06:00 h and at a temperature maintained at 20–21°C and at humidity of approximately 40%. All animals had free access to standard pellet food and water before assignment to experimental groups. The animals weighed 250–275 g at the time of surgery and 400–450 g at the time of drug testing. Additional enrichment and welfare were provided (Enviro-dri, PharmaServ) before and after the surgery. Animal health was monitored daily by the animal care staff. Surgeries were performed under ketamine and xylazine or under isoflurane anesthesia, and all efforts were made to minimize suffering. Sacrifice were done with CO_2_ or when necessary by exsanguination.

### 6-OHDA lesion

To protect norepinephrinergic neurons, animals were administered imipramine HCl (Sigma) 15 minutes before surgery. They were subsequently anesthetized with ketamine (Ceva, 75 mg/kg) and xylazine (Bayer, 10 mg/kg) and placed in stereotaxic frame (David Kopf Instrument). 6-OHDA was injected into the right ascending medial forebrain bundle at the following coordinates (in mm) relative to bregma and surface of the dura, AP = −3.5, ML = −1.5, DV = −8.7. Each rat received one injection of 6-OHDA (4 µg/µl) over a period of 5 minutes (0.5 µl/min) for a total of 10 µg per rat. Animals were monitored for 3 weeks to ensure full recovery and habituation to the environment and experimenters.

**Figure 1:**
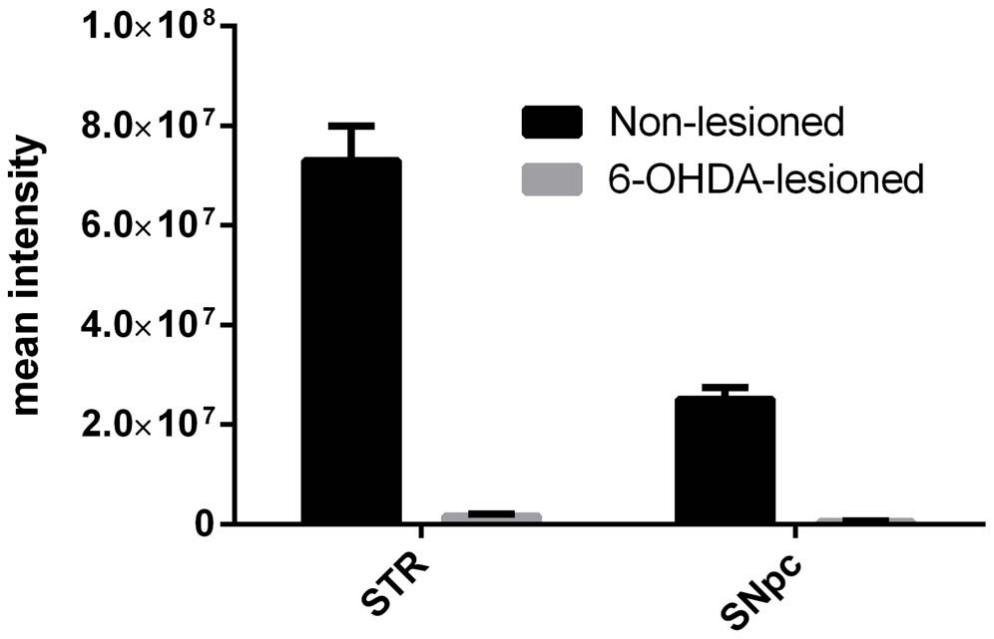
Quantification of TH and NeuN in the dopaminergic system after 6-OHDA lesioning. Intensity of TH and NeuN positive immunostaining of the substantia nigra pars compacta (left) and striatum (right) in rats (n = 24) following administration of 10 µg of 6-OHDA in the medial forebrain bundle of the right hemisphere. Immunostaining was performed in rats having received vehicle treatment only.

On day 21 post surgery, all rats were challenged with a small dose of subcutaneously administered apomorphine (Sigma, 0.05 mg/kg). Rats showing more than 90 contralateral rotations (360°) over a 45-minute recording period were included in the study. Rats meeting this criteria have a unilateral loss of dopaminergic neurons and a unilateral depletion of striatal DA of over 95% and this was demonstrated with the vehicle-treated 6-OHDA-lesioned rats which showed strong degeneration of the right dopaminergic nigrostriatal system. Internal quantification of vehicle-treated controls groups demonstrated a loss of 98% of TH immunostaining within both the striatum and the SNc (**Fig. 1**). For each experiment, rats were allocated to the different experimental groups in order to get the same mean level of basal activity measured at the apomorphine challenge amongst the different experimental groups.

### Drugs

A_2A_ and NR2B antagonist reference compounds were dissolved in a volume of 5 ml of vehicle per kg. L-Dopa methyl ester (Sigma) was dissolved in physiological saline solution at a volume of 5 ml/kg. The vehicle solution of reference compounds was made of 5% dimethyl sulfoxide (DMSO) and 95% distilled water containing 1% methyl cellulose. 6-OHDA-HBr (Sigma) was dissolved in a 0.02% ascorbic acid -distilled water at a concentration of 4 µg 6-OHDA per µl.

All compounds were obtained from commercially available standard suppliers with the exception of compound ‘Merck 22’, which was synthesized based on methodology described by [49]. The A_2A_ antagonists were Sch-58261 (Synthelabo), Preladenant (Endotherm), Tozadenant (Pharmablock) and Istradefylline (Diverchim). The NR2B antagonists were Radiprodil (Axon), Co-101244 (Key Organics) and Merck 22 (UCB synthesis).

### Behavioural recording apparatus

#### General activity in actometers (distance and rearing)

Drug-naïve separate rats were tested individually in clear acrylic chambers (LE8811 IR, Panlab). Locomotion was detected and measured by 16 infrared light-beam sensors located on each side of the enclosure at a height of 3 cm. Sensors were spaced such that the light beams formed a matrix of 15×15 squares over the surface. Activity counts were recorded on a computer and Acti-Track software (Actitrack, Panlab) was used to convert raw data into “distance traveled” and “rearing counts” for analysis as relevant dependent variables.

#### Rotational activity in rotometers (ipsi/contralateral rotations)

The rats tested one week earlier for general activity (actometers) were once again placed in clear acrylic individual chambers (50×45 cm) with the same treatment (drug and dose) than that previously received plus L-Dopa. Eight chambers were placed on a level, clear glass bottom, held by a robust frame, and were subdivided into four individual test cages of 25×22 cm. A removable acrylic plate served as a lid.

Rotational behaviour was recorded using a home-made computerized system. Rats were fixed in a harness and linked to mechanical sensors connected directly to a computer. Each 360° clock-wise or counter clock-wise turn was automatically recorded for up to 120 minutes at the maximum. Throughout the experiments, rats were allocated to individual test cages.

### Pharmacokinetics

Satellite lesioned rats (400–450 g) were anesthetized with isoflurane and sacrificed by exsanguination. Blood samples were collected in EDTA centrifuged 10 min at 3000 rpm at 4°C to obtain plasma and stored at −20°C prior the analysis. Brains were perfused by saline collected and immediately stored at −20°C prior the analysis. Brain samples were added to 4 volumes of water before being homogenized. A structural analog compound was added as internal standard to the samples and proteins were crashed using 8 volumes of acetonitrile. Compounds were analyzed on an API5000 mass spectrometer (ABSciex, Framingham, MA, USA.) equipped with a turbo ion spray source and interfaced with an Agilent 1290 Infinity LC system (Agilent Technologies, Waldbroon, Germany). The mass spectrometer was operated in the positive ionization mode with Multiple Reactions Monitoring used to quantify the various analytes (407 to 292 for Tozadenant; 398 to 109 for Radiprodil, 346 to 105 for Sch-58261 and 342 to 100 for Co-101244). A Waters HSS T3 column (2.1×30 mm, 1.8 µm) was used. The mobile phase consisted of 100% water with 0.1% formic acid (phase A) and 100% acetonitrile with 0.1% formic acid (phase B). The flow rate was 1 mL/min. A linear gradient was performed from 0 to 1 min (95 to 30% phase A), a jump to 90% B in 0.01 min, a hold period of 1.29 min at 90% phase B followed by equilibration at the initial condition was applied before the following injection.

### Experimental design

#### NR2B and A_2A_ receptor antagonist combinations as ‘monotherapy’

Six different combinations of A_2A_/NR2B antagonists were tested without L-Dopa. For each combination, there were four different procedures: vehicle only; A_2A_ antagonist followed by vehicle; vehicle followed by the NR2B antagonist and A_2A_ antagonist followed by the NR2B antagonist. The drugs, doses and combinations are described in Table 3. After drug administration, rats were placed in actometers (open-field) for monitoring the general level of motor activity (distance traveled and rearing counts). The hemilesioned rats were assessed for motor recovery in these apparatus and not in the usual rotometers (i.e. monitoring the contralateral recordings), because the behaviour displayed under the A_2A_/NR2B combination was entirely devoid of any asymmetrical bias.

**Table 3:** A_2A_ and NR2B receptor antagonist combinations tested either alone or in combination in monotherapy (i.e. without L-Dopa) in 6-OHDA-lesioned rats.

A_2A_ receptor antagonist drugs	NR2B receptor antagonist drugs
Sch-58261 1 mg/kg (ip)	Merck 22 1 mg/kg (ip)
Sch-58261 1 mg/kg (ip)	Co-101244 1 mg/kg (ip)
Preladenant 0.1 mg/kg (ip)	Merck 22 0.3 mg/kg (ip)
Preladenant 0.1 mg/kg (ip)	Radiprodil 1 mg/kg (ip)
Tozadenant 30 mg/kg (po)	Radiprodil 3 mg/kg (po)
Istradefylline 0.3 mg/kg (ip)	Co-101244 1 mg/kg (ip)

Before undertaking the study, each A_2A_ and NR2B antagonist was tested individually. The doses were selected on the basis of preliminary dose-response curves performed separately for each drug and submaximal dose (i.e. response observed is not at its maximal level) of each drug was selected for the combination studies. The two drugs were administered separately via an intraperitoneal (ip) injection with a 2-minute interval between the two drugs. Five minutes after the second injection, animals were placed in the testing cage for behavioural recording. Due to formulation and exposure issues, Tozadenant and Radiprodil had to be administered orally and therefore recording was started 60 minutes post dosing at this corresponds to maximum plasma concentrations. Eight drug-naïve animals were used for each of the four procedures; 32 in total for each combination. All behavioral experiments were conducted between 8.00 AM and 2.00 PM at the latest. Preliminary pharmacokinetic assays investigated the plasma and brain disposition of selected drugs administered alone or in combination (ie. Tozadenant/Radiprodil, and Sch-58261/Co-101244; 3 animals per group). Tozadenant and Radiprodil were measured in samples obtained 60 minutes after single oral administration at 30 mg/kg. Sch-58261 and Co-101244 were measured in samples collected 90 min after single intraperitoneal administration at 3 mg/kg.

#### NR2B and A_2A_ receptor antagonist combinations as ‘add-on therapy’ to L-Dopa

Four different combinations were administered as add-on treatment to a low active dose of L-Dopa (25 mg/kg, ip) without any dopa decarboxylase inhibitor to avoid any additional pharmacokinetic interaction. The doses of the compounds were selected on the basis of preliminary dose-response curves performed separately for each drug. To avoid ceiling effect with L-Dopa, only submaximal doses of each drug were selected for testing (as it was done previously for monotherapy). Eight L-Dopa naïve animals were tested for each of the four pharmacological condition: vehicle and L-Dopa (25 mg/kg); A_2A_ antagonist followed by vehicle and L-Dopa; vehicle followed by NR2B antagonist and L-Dopa; A_2A_ antagonist followed by NR2B and L-Dopa. Due to the dominance of the L-Dopa-effect on the lesioned striatum, the unilateral 6-OHDA-lesioned rats rotated after the drugs administration and behavioral activity was recorded in rotometers (i.e. level of contralateral rotations).

All drugs were administered i.p. 25 minutes before behavioural recording, except for the Tozadenant and Radiprodil combination which was administered orally 60 minutes before behavioural recording. As Radiprodil was administered orally, L-Dopa was given 45 minutes after the Radiprodil injection whereas for the other combinations L-Dopa was given 15 minutes after the NR2B drug. The drugs, the doses and the combinations given in co-administration to L-Dopa are described in **Table 4** **.**

**Table 4:** A_2A_ and NR2B receptor antagonist combinations tested either alone or in combination as add-on therapy to L-Dopa in 6-OHDA-lesioned rats.

A_2A_ receptor antagonist drugs	NR2B receptor antagonist drugs
Sch-58261 0.3 mg/kg (ip)	Co-101244 1 mg/kg (ip)
Preladenant 0.03 mg/kg (ip)	Radiprodil 0.3 mg/kg (ip)
Preladenant 0.03 mg/kg (ip)	Merck 22 1 mg/kg (ip)
Tozadenant 30 mg/kg (po)	Radiprodil 3 mg/kg (po)

All behavioral experiments were conducted between 8.00 AM and 2.00 PM at the latest.

### Statistics

The effects of A_2A_ and NR2B antagonists on the level of motor activity (i.e. distance traveled and rearing counts) were assessed with three-way mixed ANOVA, combining the A_2A_ receptor antagonist (2 levels, vehicle and the A_2A_ drug) and the NR2B antagonist (2 levels, vehicle and the NR2B drug) as between-group factors with the time as within-subjects factor (6 or 9 levels). The interaction between the two drugs was carefully explored, including the time effect, in order to assess whether the interaction was additive (i.e. non-significant) or synergistic (i.e. significant) to explain the increased motor activity observed overtime. To determine whether administration of the combination had any advantage over administration of single drugs, additional post hoc tests were conducted taking into account the two independent factors only. The multiple pairwise comparisons among the four different means were performed by Newman-Keuls post hoc test.

The impact of treatment with A_2A_ or NR2B receptor antagonists, given alone or in combination, on the effect of L-Dopa on contralateral rotations was also analysed with a three-way mixed ANOVA. The ANOVA included the A_2A_ and the NR2B receptor antagonists (both, 2 levels, vehicle and the drug) as between-group factors combined with time (12 levels) as within-subjects factor. Here again, the effect of the interaction between both drugs was carefully examined including the time effect to determine whether the effects observed on L-Dopa resulted from an additive (non-significant interaction between A_2A_ and NR2B drug) or a synergistic effect (significant interaction). The ability of the combination to increase and prolong the activity of L-Dopa was also explored. Consequently, drug effects were analyzed for every 10-min time interval. The reliability of the between-mean differences within the time intervals was assessed with planned contrasts using an *F* statistic. Data are expressed as averages +/- standard error of the mean. Statistical analyses were performed using the Statistica software (StatSoft Inc., OK, USA). For each test, statistical significance was assumed if P<0.05.

For every statistical analysis, a synergistic effect was concluded for the A_2A_/NR2B the combination if (1) a significant effect of the “A_2A_ xNR2B” statistical interaction was measured and, (2) if a superior effect of the group treated with the combination over the groups treated with the drugs alone was demonstrated.

## Results

### Tozadenant and Radiprodil exposure

Tozadenant and Radiprodil demonstrated similar plasma and brain exposure whether administered alone or in combination (**Table 5**). The absence of a pharmacokinetic interaction was also demonstrated for Sch-58261 and Co-101244 combination (**Table 6**). Based on literature data, the other combinations were considered unlikely to produce pharmacokinetic interactions and were thus not further assessed.

**Table 5:** Plasma and brain exposure of Tozadenant and Radiprodil administered alone or in combination.

Sample	Dosing regimen^a^	Tozadenant (µg/mL)	Radiprodil (µg/mL)
Plasma	Tozadenant	6.05±0.95	-
	Radiprodil	-	4.96±0.85
	Tozadenant + Radiprodil	5.04±0.14	5.59±2.93
Brain^b^	Tozadenant	0.18±0.04	-
	Radiprodil	-	2.63±0.56
	Tozadenant + Radiprodil	0.11±0.04	2.59±2.48

Results expressed as mean ± standard deviation of three animals.

a Both compounds were delivered orally at 30 mg/kg with samples obtained 60 min after dosing.

b Concentrations expressed per g tissue.

**Table 6:** Plasma and brain exposure of Sch-58261 and Co-101244 administered alone or in combination.

Sample	Dosing regimen^a^	Sch-58261 (ng/mL^b^)	Co-101244 (ng/mL)
Plasma	Sch-58261	97.2±10.1	-
	Co-101244	-	54.7 (n = 2)
	Sch-58261+Co-101244	112±4.16	55.4±8.32
Brain^b^	Sch-58261	246±34.7	-
	Co-101244	-	443 (n = 2)
	Sch-58261+Co-101244	264±38.3	436±74.0

Results expressed as mean ± standard deviation of three animals.

a Both compounds were delivered ip at 3 mg/kg with samples obtained 90 min after dosing.

b Concentrations expressed per g tissue.

### Impact of A_2A_/NR2B combinations on motor activity in 6-OHDA-lesioned rats

Six different combinations were tested as monotherapy (i.e. without L-Dopa): Sch-58261 + Co-101244; Preladenant + Radiprodil; Preladenant + Merck 22; Tozadenant + Radiprodil; Sch-58261 + Merck; Istradefylline + Co-101244.

A consistent, strong and lasting increase in motor activity (distance traveled and rearing counts) was observed in animals that received the combination compared with those that received single drugs, even though single drugs led to increased activity. The statistical results showed that both A_2A_ and NR2B receptor antagonist drugs had a very significant effect on the level of distance traveled and rearing counts. The analysis of the interaction of the effects between the two drugs showed that, for some combinations, the effects were only additive, when no significant interaction was found between the A_2A_ and the NR2B drugs. Whereas for other combinations, where the interaction was significant, the presence of a synergistic effect between the two compounds might be concluded.

Additional post hoc analyses, done after variance analysis, highlighted the differences between the controls (vehicle-treated rats), the rats treated with the A_2A_ antagonist or the NR2B antagonist alone, or the rats treated with the A_2A_/NR2B combination. These second-step analyses clearly demonstrated that for all the combinations, the rats treated with the A_2A_/NR2B combination had a very significantly higher level of locomotor activity than those treated with the drug alone. These results clearly demonstrated the superiority of combination treatment in hemilesioned rats.

#### Effect on distance traveled

**Figure 2:**
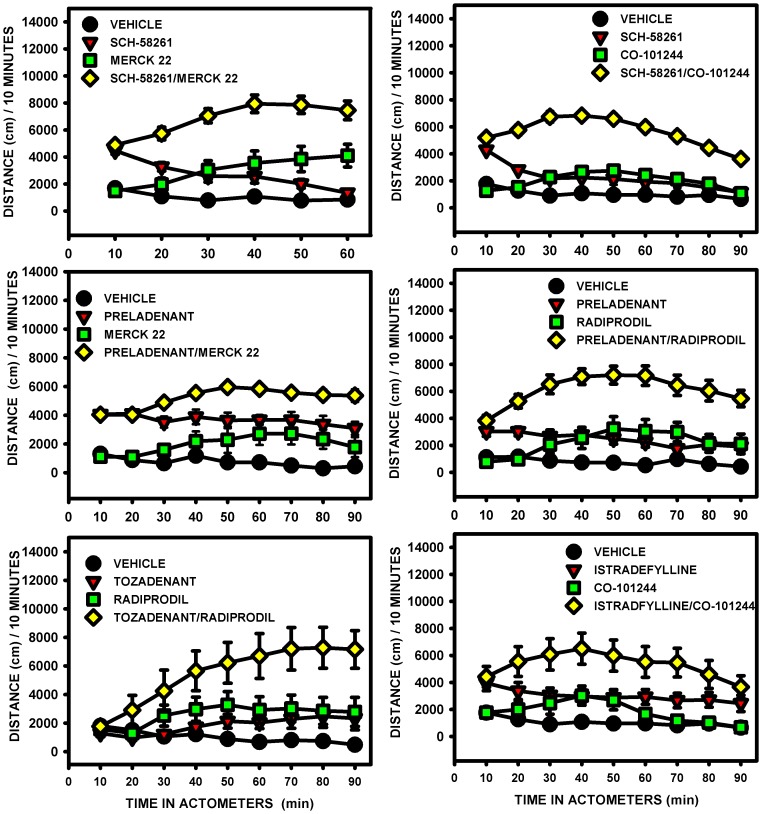
Distance traveled in unilateral 6-OHDA-lesioned rats. The effects of the A_2A_/NR2B receptor antagonist combinations, given without l-Dopa, were compared with those of the drugs alone for the distance traveled: Sch-58261 (1 mg/kg) + Merck 22 (1 mg/kg); Sch-58261 (1 mg/kg) + Co-101244 1(mg/kg); Preladenant (0.1 mg/kg) + Merck 22 (0.3 mg/kg); Preladenant (0.1 mg/kg) + Radiprodil (1 mg/kg); Tozadenant (30 mg/kg) + Radiprodil (3 mg/kg); Istradefylline (0.3 mg/kg) + Co-101244 (1 mg/kg). Administration of the combinations resulted in significantly greater distance traveled than administration of the drugs alone (Table 7; Three-way mixed ANOVA followed by Newman-Keuls post hoc test).

All A_2A_ and NR2B receptor antagonists combinations significantly increased distance traveled by the animals compared with the drug administered alone at the same dose (Fig. 2). The three-way mixed ANOVA showed a significant main effect of the A_2A_ antagonist drug, of the NR2B antagonist drug and, of time for all the combinations. For some combinations, significant “A_2A_×NR2B” interaction was also found: Sch-58261+ Merck 22; Sch-58261+ Co-101244; Preladenant + Radiprodil (Table 7). For these three specific combinations, the A_2A_ and the NR2B antagonist drugs interacted in a synergistic manner whereas in the absence of statistical significance, the interaction could be considered additive.

**Table 7:** Statistical analysis for the distance traveled following administration of A_2A_ and NR2B receptor antagonist combinations.

	A_2A_ antagonist	NR2B antagonist	A_2A_×NR2B interaction	Comment
	**Sch-58261**	**Merck 22**	**Combination**	*Synergistic effect*
Drugs main effect^a^	p<0.001	p<0.001	p<0.05	*between*
Drugs × Time effect a	Not significant	p<0.001	p<0.05	*Sch-58261*
Combination *vs* drugs alone^b^			Both p<0.001	*and*
Drugs alone *vs* vehicle^b^			Both p<0.01	*Merck 22*
	**Sch-58261**	**Co-101244**	**Combination**	*Synergistic effect*
Drugs main effect^a^	p<0.001	p<0.001	p<0.001	*between*
Drugs × Time effect a	p<0.001	p<0.001	Not significant	*Sch-58261*
Combination *vs* drugs alone^b^			Both p<0.001	*and*
Drugs alone *vs* vehicle^b^			Both p<0.01	*Co-101244*
	**Preladenant**	**Merck 22**	**Combination**	*Additive effect*
Drugs main effect^a^	p<0.001	p<0.001	Not significant	*between*
Drugs × Time effect a	Not significant	p<0.001	Not significant	*Preladenant*
Combination *vs* drugs alone^b^			Both p<0.01	*and*
Drugs alone *vs* vehicle^b^			Preladenant: p<0.001	*Merck 22*
			Merck 22: p<0.05	
	**Preladenant**	**Radiprodil**	**Combination**	*Synergistic effect*
Drugs main effect^a^	p<0.001	p<0.001	p<0.05	*between*
Drugs × Time effect a	Not significant	p<0.001	Not significant	*Preladenant*
Combination *vs* drugs alone^b^			Both p<0.001	*and*
Drugs alone *vs* vehicle^b^			Both p<0.05	*Radiprodil*
	**Tozadenant**	**Radiprodil**	**Combination**	*Additive effect*
Drugs main effect^a^	p<0.05	p<0.01	Not significant	*between*
Drugs × Time effect a	p<0.001	p<0.001	Not significant	*Tozadenant*
Combination *vs* drugs alone^b^			Both p<0.01	*and*
Drugs alone *vs* vehicle^b^			Not significant	*Radiprodil*
	**Istradefylline**	**Co-101244**	**Combination**	*Additive effect*
Drugs main effect^a^	p<0.001	p<0.05	Not significant	*between*
Drugs × Time effect a	Not significant	p<0.001	Not significant	*Istradefylline*
Combination *vs* drugs alone^b^			Both p<0.05	*and*
Drugs alone *vs* vehicle^b^			Not significant	*Co-101244*

a Primary statistical analyses conducted via Three-way mixed ANOVA;

b Post hoc analyses conducted via Newman-Keuls test.

Post hoc analyses were conducted to compare the difference between the mean of the total overall scores of the four groups: vehicle, A_2A_ antagonist, NR2B antagonist and the A_2A_/NR2B combination. The results showed that, for all combinations, the effects of drugs when administered in combination were greater than when administered alone. However, not all drugs when administered alone improved distance traveled compared with vehicle, these included Istradefylline, Tozadenant, Radiprodil (po) and Co-101244 (in one data set only).

#### Effect on rearing counts

**Figure 3:**
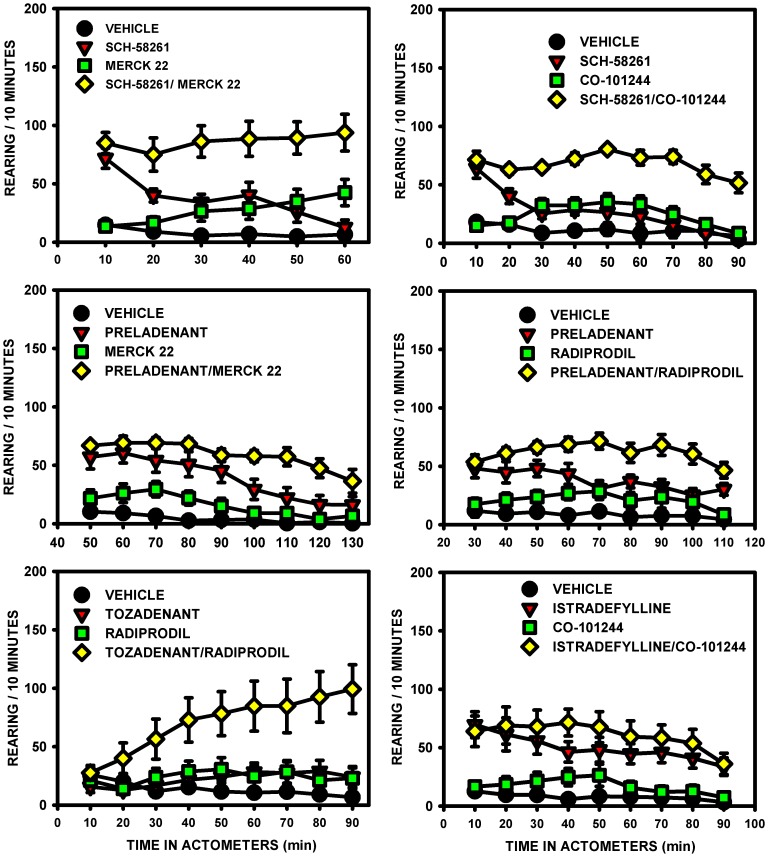
Rearing counts in unilateral 6-OHDA-lesioned rats. The effects of the A_2A_/NR2B receptor antagonist combinations, given without l-Dopa, were compared with those of the drugs alone for the number of rearing counts: Sch-58261 (1 mg/kg) + Merck 22 (1 mg/kg); Sch-58261 (1 mg/kg) + Co-101244 (1 mg/kg); Preladenant (0.1 mg/kg) + Merck 22 (0.3 mg/kg); Preladenant (0.1 mg/kg) + Radiprodil (1 mg/kg); Tozadenant (30 mg/kg) + Radiprodil (3 mg/kg); Istradefylline (0.3 mg/kg) + Co-101244 (1 mg/kg). Administration of the combinations resulted in significantly greater rearing counts than administration of the drugs alone (Table 8; Three-way mixed ANOVA followed by Newman-Keuls post hoc test).

The combination of A_2A_ and NR2B antagonist drugs was always more efficacious on the rearing counts than any individual compound administered at the same dose (Fig. 3).

Three-way mixed ANOVA showed significant main effect of A_2A_ antagonists, of NR2B antagonists and, of time (**Table 8**). However, a significant “A_2A_×NR2B” interaction was only found for the Sch-58261+ Co-101244 combination and for the triple “Preladenant × Merck 22× Time” interaction. Marginally significant effects of the interaction was also reported for two additional combinations: Sch-58261+ Merck 22 (p = 0.07) and Tozadenant + Radiprodil (p = 0.06). As above, lack of statistical significance indicates potential additive effects between the two drugs in increasing the level of rearing counts.

**Table 8:** Statistical analysis for the rearing counts following administration of A_2A_ and NR2B receptor antagonist combinations.

	A_2A_ antagonist	NR2B antagonist	A_2A_×NR2B interaction	Comments
	**Sch-58261**	**Merck 22**	**Combination**	*Additive effect*
Drugs main effect^a^	p<0.001	p<0.001	Not significant (p = 0.07)	*between*
Drugs × Time effect a	p<0.001	p<0.001	Not significant	*Sch-58261 and*
Combination *vs* drugs alone^b^			Both p<0.001	*Merck 22*
Drugs alone *vs* vehicle^b^			Sch-58261 p<0.05	
			Merck 22, not significant	
	**Sch-58261**	**Co-101244**	**Combination**	*Synergistic effect*
Drugs main effect^a^	p<0.001	p<0.001	p<0.001	*between*
Drugs × Time effect a	p<0.001	p<0.001	p<0.05	*Sch-58261*
Combination *vs* drugs alone^b^			Both p<0.001	*and Co-101244*
Drugs alone *vs* vehicle^b^			Both p<0.05	
	**Preladenant**	**Merck 22**	**Combination**	*Synergistic effect*
Drugs main effect^a^	p<0.001	p<0.01	Not significant	*between*
Drugs × Time effect a	p<0.001	Not significant	p<0.01	*Preladenant*
Combination *vs* drugs alone^b^			Both p<0.01	*and Merck 22*
Drugs alone *vs* vehicle^b^			Preladenant p<0.001	*when*
			Merck 22, not significant	*time included*
	**Preladenant**	**Radiprodil**	**Combination**	*Additive effect*
Drugs main effect^a^	p<0.001	p<0.001	Not significant	*between*
Drugs × Time effect a	Not significant	p<0.01	Not significant	*Preladenant*
Combination *vs* drugs alone^b^			Both p<0.01	*and Radiprodil*
Drugs alone *vs* vehicle^b^			Preladenant p<0.001	
			Radiprodil, not significant	
	**Tozadenant**	**Radiprodil**	**Combination**	*Additive effect*
Drugs main effect^a^	p<0.01	p<0.01	Not significant (p = 0.06)	*between*
Drugs × Time effect a	p<0.001	p<0.001	Not significant	*Tozadenant*
Combination *vs* drugs alone^b^			Both p<0.01	*and*
Drugs alone *vs* vehicle^b^			Not significant	*Radiprodil*
	**Istradefylline**	**Co-101244**	**Combination**	*Additive effect*
Drugs main effect^a^	p<0.001	Not significant	Not significant	*between*
Drugs × Time effect a	p<0.01	p<0.01	Not significant	*Istradefylline*
Combination *vs* drugs alone^b^			Co-101244, p<0.01	*and*
Drugs alone *vs* vehicle^b^			Istradefylline, p<0.01	*Co-101244*
			Co-101244, not significant	

a Primary statistical analyses conducted via Three-way mixed ANOVA;

b Post hoc analyses conducted via Newman-Keuls test.

After the variance analyses, Newman-Keuls post hoc tests were conducted on the total rearing scores to compare the effects of the four different treatments: vehicle, A_2A_ receptor antagonist drug, NR2B receptor antagonist drug and the combination. Results showed that the effects of all drugs were greater when administered in combination than when administered alone, with the exception of the “Istradefylline + Co-101244” combination, where its effect was only superior to Co-101244 but not to Istradefylline (**Table 8**).

Results also showed that not all NR2B receptor antagonists tested alone (Merck 22, Radiprodil and Co-101244 in one set of experiment) increased the number of rearing counts in comparison to vehicle rats, whereas all the A_2A_ receptor antagonists did, with the exception of Tozadenant.

### A_2A_/NR2B combination prolongs L-Dopa effect on contralateral rotations

**Figure 4:**
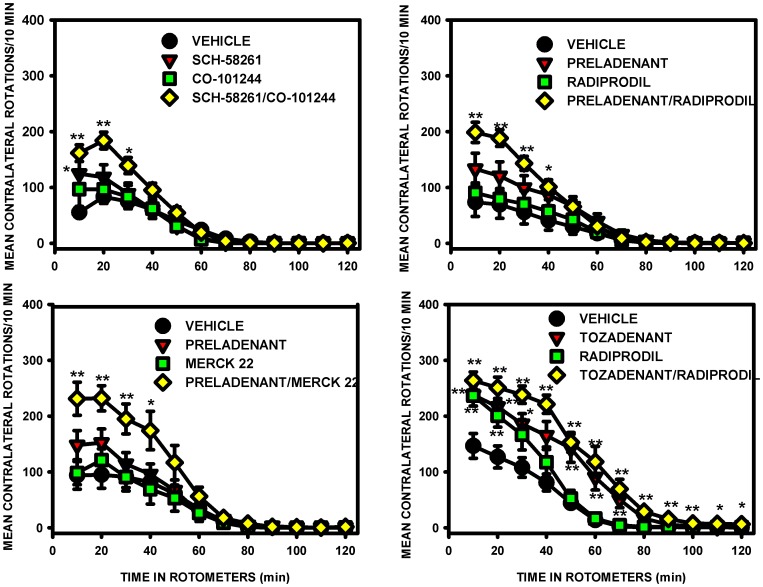
L-Dopa-induced contralateral rotations in unilateral 6-OHDA-lesioned rats. The effects of the A_2A_/NR2B receptor antagonist combinations, given with L-Dopa, were compared with those of the antagonists alone for the number of contralateral rotations: Sch-58261 (0.3 mg/kg) + Co-101244 (1 mg/kg); Preladenant (0.03 mg/kg) + Radiprodil (0.3 mg/kg); Preladenant (0.03 mg/kg) + Merck 22 (1 mg/kg); Tozadenant (30 mg/kg) + Radiprodil (3 mg/kg). All combinations showed significant greater contralateral rotations than vehicle for the respective time intervals (Table 9; Three-way ANOVA and planned contrasts [*p<0.05 and **p<0.01 versus vehicle group]).

Four different combinations were tested as add-on therapy to L-Dopa: Sch-58261+ Co-101244; Preladenant + Radiprodil; Preladenant + Merck 22; Tozadenant + Radiprodil (**Fig. 4**). Due to the dominance of L-Dopa in the lesioned striatum, the hemilesioned rats rotated and contralateral rotations were monitored in rotometers to evaluate drug effects. To avoid the ceiling effect with L-Dopa, only a sub-maximal dose of A_2A_ and NR2B receptor antagonists was used (doses were determined by previous dose-response investigations using the antagonists alone).

Three-way mixed ANOVA showed that, when given in combination with NR2B receptor antagonist, all A_2A_ receptor antagonists showed significant overall effect on the level of contralateral rotations induced by a low active dose of L-Dopa. In contrast, with the exception of Radiprodil in combination with Tozadenant, other NR2B antagonist drugs did not show any significant main effect when administered in combination. The analysis of the “A_2A_× NR2B” interaction across the four different combinations did not show any significance, suggesting that the improvement observed with the two drugs administered together, corresponded to a summation of their effect on L-Dopa rather than to a synergistic phenomenon. However, a very strong overall effect of the time was observed for the four combinations and this time effect also strongly interacted with all the A_2A_ and NR2B antagonists tested. Planned comparisons aimed at evaluating the effects of the combination with the vehicle or the effect of the drugs alone with the vehicle for each 10-min time interval. These analyses showed that the combination significantly increased and prolonged the level of L-Dopa-induced contralateral rotations compared with the drugs alone. Results also showed that the A_2A_ or NR2B antagonist, when given separately, did not increase or prolong L-Dopa effect with the exception of Tozadenant and Radiprodil which were found to be active for 80 and 20 minutes respectively (**Table 9**).

**Table 9:** Statistical analysis for the contralateral rotations following administration of A_2A_ and NR2B receptor antagonist combinations as add-on treatment to l-Dopa.

	A_2A_ antagonist	NR2B antagonist	A_2A_×NR2B interaction	Comments
	**Sch-58261**	**Co-101244**	**Combination**	*Additive effect*
Drugs main effect^a^	p<0.05	Not significant	Not significant	*between*
Drugs × Time effect^a^	p<0.001	p<0.001	Not significant	*Sch-58261*
Combination vs vehicle^b^			p<0.05 for 30′	*And*
Drugs alone vs vehicle^b^			Sch-58261, p<0.05 for 10′	*Co-101244*
	**Preladenant**	**Merck 22**	**Combination**	*Additive effect*
Drugs main effect^a^	p<0.05	Not significant	Not significant	*between*
Drugs × Time effect^a^	p<0.001	p<0.01	Not significant	*Preladenant*
Combination vs vehicle^b^			p<0.05 for 40′	*and*
Drugs alone vs vehicle^b^			Not significant	*Merck 22*
	**Preladenant**	**Radiprodil**	**Combination**	*Additive effect*
Drugs main effect^a^	p<0.01	Not significant	Not significant	*between*
Drugs × Time effect^a^	p<0.001	p<0.01	Not significant	*Preladenant*
Combination vs vehicle^b^			p<0.05 for 40′	*and*
Drugs alone vs vehicle^b^			Not significant	*Radiprodil*
	**Tozadenant**	**Radiprodil**	**Combination**	*Additive effect*
Drugs main effect^a^	p<0.001	p<0.01	Not significant	*between*
Drugs × Time effect^a^	p<0.001	p<0.001	Not significant	*Tozadenant*
Combination vs vehicle^b^			p<0.05 for 120′	*and*
Drugs alone vs vehicle^b^			Tozadenant p<0.05 for 80′	*Radiprodil*
			Radiprodil p<0.05 for 20′	

a Primary statistical analyses conducted via Three-way mixed ANOVA;

b A priori Planned Contrasts done on each.

10-min time interval of rotations recording.

## Discussion

A_2A_ and NR2B receptor antagonists both show anti-parkinsonian activity in preclinical models; however, the effects are weaker in magnitude compared with dopamine agonists or L-Dopa [14]–[16], [34]. A_2A_ antagonists have also demonstrated weak efficacy in clinical trials when tested as monotherapy in PD patients [22]. Given the localization of A_2A_ and NR2B receptors within the basal ganglia and the numerous lines of evidence which suggest that they interact on a molecular level, we wanted to test if NR2B and A_2A_ antagonist combination treatment might indeed provide increased efficacy in PD models. The results obtained show that concomitant administration of a NR2B receptor antagonist with an A_2A_ receptor antagonist substantially increased the quantity of motor activity in hemi-lesioned rats in comparison to the effects measured with the compounds alone. Interestingly, the observed motor activity did not consist of a rotational response as seen with dopaminergic treatment but rather evoked straight displacement in the absence of any body torsion which suggests a normalization of the movement trajectory and of the body position. Furthermore, the activity observed with some combinations could not be described by a simple additive effect and might involve more subtle pharmacodynamics synergistic effect since significant interaction was found between some A_2A_ and the NR2B antagonist drugs.

Each of the A_2A_ antagonists tested alone gave a moderate increase in the distance traveled by the lesioned rats although this did not always reach statistical significance. For example, rats treated with Sch-58261 and Preladenant showed significant increase of the distance traveled in comparison to vehicle-treated controls but not those treated with Tozadenant or Istradefylline. Several studies have shown the ability of A_2A_ antagonists to ameliorate the parkinsonian motor symptoms when given as monotherapy (i.e. without L-Dopa) in preclinical models. A_2A_ antagonists have been shown to be efficacious against haloperidol-induced catalepsy [16], [20], [50] and showed positive effects in the forepaw stepping test in unilaterally 6-OHDA lesioned rats [51] and in the rotarod test [52]. However, most studies failed to demonstrate a significant effect of A_2A_ receptor antagonists on the level of contralateral rotation in lesioned rats in the absence of L-Dopa [14]–[16], [53]. To our knowledge, this is the first time that the efficacy of A_2A_ receptor antagonists on motor activity has been systematically evaluated in unilaterally 6-OHDA-lesioned rats by measuring the general level of motor activity. Most remarkably, A_2A_ antagonists not only (in some instances markedly) increased the motor activity of the animals without inducing rotation, but also, with the exception of Tozadenant, increased the level of rearing, a behaviour which is typically lost following administration of MPTP and 6-OHDA [54].

The NR2B antagonists only weakly increased motor activity, with only Merck 22, Co-101244 and Radiprodil (ip) increasing the distance traveled to a statistically significant level. NR2B receptor antagonists did not increase the level of rearing in unilateral 6-OHDA-lesioned rats with the exception of Co-101244.

These data are in line with reports in the literature showing that NR2B antagonists are able to improve some parkinsonian symptoms. However, different tests were used and to our knowledge no data exist demonstrating the activity of such a treatment in the open-field. The non-selective NMDA antagonists CPP and dizocilpine have been shown to ameliorate the stepping deficit in rats with a unilateral lesion of the medial forebrain bundle [55]. The NR2B selective antagonist Ifenprodil was shown to counteract Reserpine-induced akinesia at the highest dose tested [35]. Loschmann et al. reported the efficacy of Ro 25–6981 in inducing contralateral rotations in unilaterally 6-OHDA-lesioned rats when given as monotherapy and tested in a rotometer [34]. In agreement with this finding, we also observed increased contralateral rotations with some NR2B receptor antagonists when administered as monotherapy and tested for rotations in rotometers [56].

Careful observation of the animals in different environments led us to pursue the investigations in the open-field instead of narrow rotometers, when the drugs where given without L-Dopa (i.e. in monotherapy). By allowing the animals to move freely in a larger space, NR2B or A_2A_ receptor antagonists were found to be more active than previously reported, with the animals showing exploratory and thigmotactic behaviour with straight movements. More importantly, as opposed to what is typically observed in unilaterally-lesioned rats receiving a dopaminergic treatment (L-Dopa or dopamine agonists), no behavioural asymmetry was observed with these drug treatments.

Although the locomotor activities induced by the individual compounds were significant in some cases, combined treatment with A_2A_ and NR2B receptor antagonists clearly showed major additional improvement in motor function in unilateral 6-OHDA-lesioned rats. This increased efficacy might be attributed to an additive effect or to a synergistic effect using different combinations of the different compounds. In any case, the observed improvements were strong, highly reproducible and long-lasting. For every group treated with the A_2A_/NR2B combination, we consistently observed a significant increase in both the distance traveled and the number of rearing counts compared with vehicle-treated animals but also with animals treated with the same dose of each drug alone. In addition to the quantity of movement quantified automatically, visual observation of the rats in the activity chambers showed a surprising richness of behaviour. Furthermore, besides the strong increase of motor activity, the animals did not exhibit the typical contralateral rotations observed with stimulant drugs such as dopaminergic agents [57], [58]. They also did not show solely ipsilateral rotations as observed with dopamine transporter blockers [59], the dopamine releaser amphetamine [48] and with 5-HT1A agonists [60]. The locomotor activity was accompanied by thigmotactic behaviour, as well as centre exploration. During movements, the animals switched from one direction to the other (left to right and right to left) spontaneously and covered the entire surface of the open-field while exploring. In addition, all these behaviours were performed with an adequate quadrupedal and straight body position seemingly quite similar to normal body positioning and movement (data on file, detailed report in preparation). No abnormal involuntary or stereotypic movements were observed. These are all behavioural characteristics that had never been observed together with dopaminergic drugs. In addition, this behavioral assessment would never have been possible with the traditional measure of motor recovery, i.e.contralateral rotations, which only records the motor stimulation produced by dopaminergic drugs.

Previously, one report demonstrated that the co-administration of CGP37849, a competitive, and Dizocilpine, a non-competitive, NMDA receptor antagonist could potentiate the effects of a threshold dose of Theophylline, a non-selective adenosine receptor antagonist, in the model of haloperidol-induced catalepsy [61]. In addition, the potential benefits on the restoration of a more complex behaviour could not be monitored since the behavioural assessment only corresponded to a measure of the latency time for catalepsy.

Preliminary investigations were performed to determine whether the improvement in motor activity following administration of the combinations might be due to pharmacokinetic interactions between the two drugs. When co-administered, the respective plasma and brain levels of Tozadenant and Radiprodil were not different from those observed when the compounds were administered alone. Lack of pharmacokinetic interaction was also demonstrated for the Sch-58261 and Co-101244 combination. Although not assessed, pharmacokinetic interaction with the other combinations is unlikely based on literature data. Preladenant was reported to be devoid of *in-vitro* risks of drug interactions with cytochrome P-450 (CYP) and P-glycoprotein (Pgp) substrates, a finding confirmed in clinical studies [62]. Absence of *in-vitro* interactions with CYPs and Pgp was also reported for the NR2B antagonist Merck 22 compound [49]. Finally, in human subjects, Istradefylline does not impair CYP3A-mediated drug metabolism, its effects being restricted to modest inhibition of P-gp [63]. Overall, it is reasonable to conclude that the synergistic activity observed with some of the A_2A_ and NR2B combinations is unlikely to result from changes in drug disposition.

To interpret the behavioural observations, one might speculate that the improvement and richness of the locomotor activity observed under the A_2A_ and NR2B combination, including the absence of behavioral asymmetry, is due to a restoration of the balance of the dysregulated circuits of the basal ganglia. This could be due to a differential but unique stimulation of both the direct and indirect pathways by the two compounds, mediated by the specific receptor distributions and their effects at different sites of the circuits. That re-equilibration might be expected to mainly result from the direct inhibition of the overactive indirect striatopallidal pathway. However, NR2B antagonism could also modulate the glutamatergic cortical input to the striatum [29] while inhibition of pre-synaptic A_2A_ receptors present in glutamatergic terminals of cortico-striatal afferents to the direct MSN could further reduce the release of glutamate into the striatum [64] . Dampening this activity would additionally decrease the over-activity of the indirect striatopallidal pathway. These mechanistic hypotheses strongly deserve further investigation.

In addition to the effects of the combined administration of an NR2B receptor antagonist and an A_2A_ receptor antagonist on the locomotor activity in unilateral 6-OHDA lesioned rats, our data also showed that co-administration of the A_2A_/NR2B combination is able to potentiate L-Dopa efficacy (add-on therapy). Due to the dominance of L-DOPA in the basal ganglia, the animals rotated and were monitored in rotometers in contrast to the previous investigations. Surprisingly, the addition of either an A_2A_ receptor antagonist or of an NR2B receptor antagonist alone did not increase and/or prolong the effect of L-Dopa on the level of contralateral rotation. These observations contrast with literature reports [34], [37], [65] but may reflect the fact that lower doses of A_2A_ and NR2B antagonists were used in our study. Despite the lack of efficacy when the compounds were administered alone with L-Dopa, the combined administration of an A_2A_ and a NR2B receptor antagonist to the same dose of L-DOPA significantly increased the magnitude of the L-Dopa effect on the level of contralateral rotations. This observation suggests that the drug effects should be translatable to the clinical condition: in that, the addition of A_2A_ antagonists to L-DOPA has been shown to enhance the efficacy of L-DOPA as measured as a reduction in off-time. Current reviews confirm that the level of contralateral rotations and the ability of compounds to increase this parameter are a reliable index for the clinical activity of the antiparkinsonian potential of newly developed drugs [66].

In conclusion, in the present study we have demonstrated that the combined administration of an NR2B receptor antagonist with an A_2A_ receptor antagonist in monotherapy (i.e. without L-Dopa) significantly increased both the quantity and the quality of movements in unilateral 6-OHDA-lesioned rats. Furthermore, the NR2B/A_2A_ receptor antagonist combination also significantly potentiated the efficacy of L-Dopa when given as add-on therapy. Given the accepted translatability of the 6-OHDA model we believe that such combination therapy could have a profound effect on the motor symptoms of PD patients. Our aim was to study the effect of the combination on the motor symptoms, however, it is worth mentioning that both NR2B and A_2A_ antagonists could modify non-motor symptoms. Indeed A_2A_ antagonists have been proposed to improve several non-motor symptoms such as excessive daytime sleepiness [67], cognition and depression [68], while NR2B antagonists could improve depression and anxiety [69]. Furthermore, both NR2B antagonists [70] and A_2A_ receptor antagonists [12] have been suggested to be neuro-protective through different mechanisms so, the combined use could theoretically also modify disease progression. Given the data presented here, we propose that monotherapy with a drug combining both A_2A_ and NR2B receptor antagonist properties could provide significant benefit in early or *de novo* PD patients while this combination given as add-on therapy to L-DOPA could be used in advanced and fluctuating Parkinson's disease patients.
